# HbA2-Yokoshima (delta 25(B7)Gly >Asp) and Hb A2-Yialousa (delta 27(B9)Ala>Ser) in Turkey

**DOI:** 10.5505/tjh.2012.50470

**Published:** 2012-10-05

**Authors:** Aylin Köseler, Ayfer Atalay, Erol Ömer Atalay

**Affiliations:** 1 Pamukkale University, School of Medicine, Department of Biophysics, Denizli, Turkey

## TO THE EDITOR

Heterozygous beta-thalassemia (β-thal) carriers are characterized by microcytosis, hypochromia, and elevated HbA_2_ levels (≥3.5%) [1]. Although an elevated HbA_2_ level is a diagnostic parameter for b-thal, the interaction between d-globin gene mutation and b-thal can result in a normal HbA_2_ level, leading to misdiagnosis [2]. As δ-thalassemia (δ-thal) has no clinical significance, a reduced HbA_2_ level in β-thal carriers is an important parameter in the presence of d-thalassemia [3]. δ-globin gene mutations (http://globin.cse.psu.edu/hbvar/menu.html) have been reported [4]. HbA_2_-Yialousa (delta 27(B9)Ala>Ser) is the most common δ-thal mutation in the Mediterranean Region and was first identified by Trifillis et al. in a Sardinian family in 1991 [1,5]. HbA_2_-Yokoshima (delta 25(B7)Gly >Asp) was first identified in a Japanese family in which 1 member was homozygous [6]. 

Altay et al. reported the presence of abnormal hemoglobin variants in their review, including a-globin, b-globin, and d-globin variants [7]. In 2007 Akar et al. reported that there were 88 hemoglobin variants [8]. As most hemoglobin variants are asymptomatic, they are often detected during family and population studies, and premarital screening programs. As an example, during premarital screening in Denizli, Turkey, several variants of hemoglobin were observed and identified; Hb-Yaizu, Hb-Ouled Rabah, and Hb-Tunis were the first reported cases in Turkey [9,10]. 

**Figure 1:**
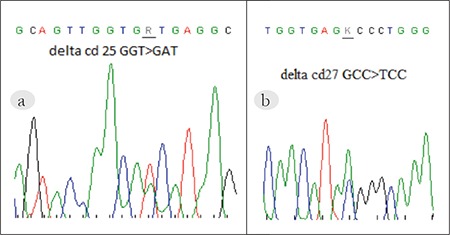
DNA sequencing of the HbA_2_-Yokoshima (a) and HbA_2_-Yialousa (b) cases.

In total, the DNA of 12 b-thalassemia carriers with a low HbA_2_ level was studied. Written informed consent was obtained from the patients during donation of their DNA for use as anonymous samples. DNA amplification and sequencing were performed using a BECKMAN Coulter CEQ8000 non-radioactive fluorescence dye-based genetic analysis system, according to Pavlou et al. [11]. We identified IVS-1/nt-6 (T>C), IVS-1/nt-110 (G>A), IVS-2/nt-1 (G>A), and Cd44 (-C) mutations in all the DNA samples. Only 3 of the 12 b-thal carriers had a low HbA_2_ level and d-globin gene mutation. DNA sequencing showed that the mutation at d-globin gene codon 25 (GGT/GAT) caused Hb-Yokoshima (Figure 1a) and mutation at d-globin gene codon 27 (GCC/TCC) caused Hb-Yialousa (Figure 1b). All of the patients that carried d-globin mutations also carried (b-thal) IVS-1-110 (G>A) mutation at the b-globin gene.

In conclusion, we would like to emphasize the importance of HbA_2_ variants in premarital diagnosis, and that the presence of variants of this δ-globin resulting in decreased HbA_2_ expression could lead to misdiagnosis of β-thal carrier status. 

## ACKNOWLEDGMENTS

We are grateful to Professor Dr. Ali Keskin from Pamukkale University, School of Medicine, Department of Hematology, and Dr. Hasan Koyuncu from the Turkish Ministry of Health, Denizli Hemoglobinopathy Center, Denizli, Turkey, for their valuable contributions.

## CONFLICT OF INTEREST STATEMENT

None of the authors has any conflicts of interest, including specific financial interests, relationships, and/or affiliations, relevant to the subject matter or materials included.
